# Low Vitamin D and High Psychological Distress: Are They Associated with Poor Differentiation in Head and Neck Cancer?

**DOI:** 10.3390/clinpract15090164

**Published:** 2025-09-12

**Authors:** Bogdan Hirtie, Ana-Maria Stanoiu, Kristine Guran, Norberth-Istvan Varga, Claudia Raluca Balasa Virzob, Delia Hutanu, Adrian Cote, Rodica Anamaria Negrean, Delia Ioana Horhat, Cristian Ion Mot

**Affiliations:** 1 Doctoral School, Department of General Medicine, “Victor Babes” University of Medicine and Pharmacy, Eftimie Murgu Square No. 2, 300041 Timisoara, Romania; bogdan.hirtie@umft.ro (B.H.); ana-maria.stanoiu@umft.ro (A.-M.S.); guran.kristine@umft.ro (K.G.); norberth.varga@umft.ro (N.-I.V.); 2 Department of Nursing, “Victor Babes” University of Medicine and Pharmacy, 300041 Timisoara, Romania; virzob.claudia@umft.ro; 3 Department Biology-Chemistry, Faculty of Chemistry-Biology-Geography, West University of Timisoara, Pestalozzi, 16, 300315 Timisoara, Romania; delia.hutanu@e-uvt.ro; 4 Department of Surgical Disciplines, Faculty of Medicine and Pharmacy, University of Oradea, 410073 Oradea, Romania; adrian.cote@didactic.uoradea.ro; 5 Department of Preclinical Disciplines, Faculty of Medicine and Pharmacy, University of Oradea, 410073 Oradea, Romania; 6 ENT Department, “Victor Babes” University of Medicine and Pharmacy, Eftimie Murgu Square No. 2, 300041 Timisoara, Romania; horhat.ioana@umft.ro (D.I.H.); mot.ion@umft.ro (C.I.M.)

**Keywords:** vitamin D, 25-OH-vitamin D, psychological distress, anxiety, depression, HADS, HNC, head and neck cancer, ENT, ears, nose and throat

## Abstract

**Background and Objectives:** Vitamin D deficiency and psychological distress have been linked to cancer biology, but their relevance to tumor differentiation in head-and-neck squamous cell carcinoma (HNSCC) is uncertain. **Materials and Methods:** In this cross-sectional study at the Department of Otolaryngology, County Hospital of Timișoara, Romania, we enrolled newly diagnosed HNC patients from October 2023 to December 2024, analyzing 199 SCC patients after exclusions. Vitamin D status was assessed using serum 25-OH-vitamin D levels, and distress was measured with the validated Romanian version of the Hospital Anxiety and Depression Scale (HADS). Tumor aggressiveness was defined by histological grade (G3 vs. G1–G2). Univariate, multivariate, and subgroup analyses were conducted, adjusting for confounders like smoking. **Results:** Vitamin D deficiency (<20 ng/mL) was prevalent (80.40%), with median 25-OH-vitamin D levels of 15.1 ng/mL. Univariate analysis revealed a modest association between vitamin D deficiency and poorly differentiated tumors (G3 vs. G1–G2; OR = 1.79, *p* = 0.055) and between clinically significant anxiety (HADS-A ≥ 8) and G3 tumors (OR = 1.71, *p* = 0.059). A weak negative correlation was observed between 25-OH-vitamin D levels and HADS-A scores (rho = −0.17, *p* = 0.052). In multivariate analysis adjusted for age, smoking, and tumor location, these associations weakened (vitamin D deficiency: OR = 1.55, *p* = 0.082; HADS-A ≥8: OR = 1.56, *p* = 0.113). Subgroup analysis suggested a trend toward higher odds of G3 tumors in patients with both vitamin D deficiency and high anxiety (OR = 1.72, *p* = 0.075). **Conclusions**: Univariate analyses indicated potential links between vitamin D deficiency, psychological distress, and tumor aggressiveness in HNSCC, but these did not reach statistical significance after adjustment for confounders. The observed trends, particularly in subgroups with combined deficiency and distress, suggest a possible interplay worth exploring further. To conclude, neither vitamin-D deficiency nor clinically significant distress independently predicted poor histological differentiation after adjustment; observed trends, including a possible distress–vitamin-D interaction, are hypothesis-generating and warrant testing in larger, longitudinal cohorts.

## 1. Introduction

Head and neck squamous cell carcinoma (HNSCC) remains a major oncological challenge, accounting for about 890,000 new cases and 450,000 deaths worldwide each year [1]. Despite multimodal therapy, population–based analyses show that five-year survival for HNSCC has crept upward by substantially less than 1 percentage point per year over the past two decades [2], underscoring the need to identify modifiable biological and psychosocial factors that might influence tumor behavior at presentation as well as long-term outcome. Over the past two decades, 5-year survival for head and neck squamous cell carcinoma (HNSCC) has risen from ~55% in the 1990s to ~68% in the 2010s; contemporary SEER summaries place overall 5-year survival near 68% (2018 cohort) [3,4,5].

Vitamin D deficiency is common in patients with solid tumors, including HNSCC, where reported prevalence ranges from 47% to 95% [6]. After cutaneous ultraviolet-B exposure or oral intake, vitamin D is converted to its circulating pro-hormone 25-hydroxyvitamin D (25-OHD) and, subsequently, to the active metabolite 1,25-dihydroxyvitamin D (calcitriol). Calcitriol binds the vitamin-D receptor (VDR), which is expressed in head-and-neck epithelia and immune cells, triggering transcriptional programs that promote apoptosis, inhibit angiogenesis, and temper nuclear-factor-κB (NF-κB)-driven inflammation [5,6]. Low serum 25-OHD has been linked to increased cancer incidence, larger primary tumors, and reduced overall survival in HNSCC cohorts [7,8,9]; however, its association with histological grade at diagnosis—a surrogate of intrinsic tumor aggressiveness—has not been explored systematically.

Psychological distress, encompassing clinically significant anxiety and depression, affects up to two-thirds of patients with newly diagnosed HNSCC [10,11]. The Hospital Anxiety and Depression Scale (HADS) remains the most widely adopted screening tool in this setting; scores ≥8 on either subscale indicate symptoms that may warrant intervention [12]. Distress has been linked to poorer quality of life, reduced treatment adherence, higher loco-regional relapse rates and shorter overall survival in patients with HNSCC [13,14]. Mechanistically, activation of the hypothalamic–pituitary–adrenal axis and sympathetic nervous system elevates cortisol and catecholamines, sustaining NF-κB-mediated transcription of pro-inflammatory cytokines and impairing cytotoxic T-cell and natural-killer-cell function [15,16].

Both vitamin D deficiency and chronic psychological distress converge on the tumor–immune micro-environment, tipping the balance toward immune evasion and aggressive growth. Pre-clinical work shows that calcitriol can dampen stress-induced inflammatory signaling, while stress hormones can down-regulate VDR expression, suggesting bidirectional crosstalk [17,18]. These shared pathways raise the possibility that concomitant deficiency and distress might exert additive or even synergistic effects on tumor differentiation.

Systematic reviews have reported modest inverse correlations between circulating 25-OHD concentrations and anxiety scores in both community and clinical samples [19]. Proposed mechanisms include vitamin D-dependent regulation of serotonergic neurotransmission and neuroinflammation. Whether such an interaction is relevant in the specific context of HNSCC, where both deficiency and distress are highly prevalent, remains unclear. Observational studies in HNSCC report that lower serum 25-hydroxyvitamin D associates with adverse pathological features and poorer overall survival, while higher vitamin-D exposure metrics relate to more favorable outcomes. In parallel, histological grade retains independent prognostic value in oral cavity and other HNSCC subsites [20,21].

To our knowledge, no clinical study has simultaneously examined the relationships among vitamin D status, psychological distress, and histological grade at first presentation of HNSCC. Therefore, we aimed to test the hypothesis that: (i) serum 25-OHD < 20 ng mL^−1^, and (ii) clinically meaningful distress (HADS-A or HADS-D ≥8), are each independently associated with poor tumor differentiation (grade 3) at diagnosis, after adjustment for age, smoking intensity, and tumor sub-site. Clarifying these associations could inform risk stratification and support integrated nutritional and psycho-oncological interventions in routine HNSCC care.

## 2. Materials and Methods

### 2.1. Study Design and Population

This cross-sectional analysis was conducted within a cohort of patients diagnosed with ENT (ear, nose, and throat) cancers at the Department of Otolaryngology, County Hospital of Timișoara, Romania. Adult patients (aged 18 years or older) newly diagnosed with cancer in the ENT sites (malignancies of the oral and nasal cavities, naso-, oro-, and hypo-pharynx, larynx, major salivary glands) were enrolled between 1 October 2023, and 30 December 2024. Patients were considered eligible if baseline assessments, including the Hospital Anxiety and Depression Scale (HADS), were completed at the time of diagnosis, and data on 25-OH-vitamin D levels and tumor characteristics were available. Exclusion criteria were the following: (i) age under 18, (ii) in situ carcinomas and non-Hodgkin Lymphomas, (iii) patients with cognitive impairment that prevented participation (cognitive impairment was defined as inability to understand and/or answer the questionnaire, as determined by a clinical interview with the hospital psychiatrist), and (iv) patients who declined to provide informed consent.

A power analysis was conducted in order to estimate the number of patients that should be enrolled. To detect an odds ratio of 2.0 for the association between vitamin-D deficiency or elevated psychological distress (HADS ≥ 8) and poorly differentiated tumors (G3), we assumed a 70% exposure prevalence and a 10% G3 prevalence. Using G*Power 3.1.9.7 (two-tailed α = 0.05, power = 0.80), ≥200 participants were required; therefore 240 were targeted to allow for 20% exclusions.

### 2.2. Data Collection

Data were extracted retrospectively from medical records and study-specific questionnaires completed at the time of diagnosis, between1 October 2023, and 30 December 2024. Baseline assessments were performed at the point of initial diagnosis, typically during hospitalization for diagnostic confirmation or treatment planning. Demographic, clinical variables, and medical history including age, sex, tumor location, smoking status, alcohol use, BMI, prior vitamin D supplementation, prior anti-depression and/or anti-anxiety medication, and comorbidities were collected at baseline. For solid tumors in the ENT region, TNM stage (I–IV) and histological differentiation (G1–G3) were noted. Stage was excluded as an outcome because it reflects diagnostic delay as well as tumor biology. Our aim was to test intrinsic aggressiveness measurable on the initial biopsy. Tumor staging and grading were conducted according to the American Joint Committee on Cancer (AJCC) 8th edition criteria, ensuring standardized classification [5]. Histological grade was assigned on diagnostic H-E slides by two pathologists from our hospital: (1) G1 = well-differentiated keratinising; (2) G2 = moderately differentiated; and (3) G3 = poorly differentiated/non-keratinising. Discrepancies were resolved by joint review. Non-Hodgkin lymphomas (NHL) presenting in ENT sites were excluded from the analysis, because their lymphoid histogenesis, staging system, tumor-grade criteria differ fundamentally from those of mucosal squamous-cell carcinomas.

Psychological distress was assessed using the Hospital Anxiety and Depression Scale (HADS), a 14-item self-report questionnaire with two subscales for anxiety (HADS-A) and depression (HADS-D). The HADS questionnaire was administered in its previously validated Romanian version, following the same methodology as Hirtie et al. (2025) [15], conducted in the same hospital setting. Each subscale ranges from 0 to 21, with scores ≥8 indicating clinically significant symptoms. Prior to HADS administration, cognitive capacity was screened by the hospital psychiatrist through a brief clinical interview. This assessment ensured that participants could comprehend the questionnaire instructions and provide reliable responses, in line with the study’s inclusion criteria. The HADS questionnaire was distributed to patients in a quiet, private setting during their hospital visits. Participants were given clear verbal and written instructions to complete the questionnaire independently, ensuring they understood each item’s response options (scored on a 4-point Likert scale from 0 to 3). Completed questionnaires were collected immediately by research staff to prevent loss and ensure data integrity. This process was designed to minimize response bias and ensure accurate self-reporting of anxiety and depression symptoms. The Romanian HADS shows good internal consistency (Cronbach’s α = 0.84 for HADS-A and 0.82 for HADS-D) and test–retest reliability (ICC = 0.78–0.83), reproducing the original two-factor structure [15].

Serum 25-OH-vitamin D levels were measured at diagnosis using the Alinity m 25-OH Vitamin D assay (Abbott Laboratories, Des Plaines, IL, USA), a chemiluminescent microparticle immunoassay (CMIA) with a sensitivity of 4 ng/mL and inter-assay coefficient of variation <10%. Results were expressed in ng/mL, with vitamin D status categorized as deficient (<20 ng/mL), insufficient (20–29.9 ng/mL), or sufficient (≥30 ng/mL), based on established clinical thresholds. HPV status was assessed for all patients using the Alinity m HR HPV assay (Abbott Laboratories), a qualitative real-time PCR-based test targeting 14 high-risk HPV genotypes. Mucosal specimens were collected via endocervical or oropharyngeal swabs in ThinPrep PreservCyt Solution. All data were de-identified for analysis. Only research team members involved in data collection (B.H., D.I.H., and C.I.M.) had access to patients’ full names, while others worked with initials to ensure confidentiality.

### 2.3. Ethical Considerations

The study adhered to the principles of the Declaration of Helsinki and was approved by the Ethics Committee of the Timișoara County Hospital (protocol code 71/02.10.2023, date 2 October 2023). Written informed consent was obtained from all participants prior to data collection, ensuring that the study’s purpose and their rights were understood. Patient confidentiality was strictly maintained throughout the research process. Generative AI (ChatGPT v4.0, OpenAI, San Francisco, CA, USA) was used solely with the purpose of checking grammar and language editing, while the data collection process, statistical analysis, interpretation, and reporting of findings, were performed exclusively by the authors.

### 2.4. Statistical Analysis

Statistical analyses were performed using SPSS version 27 (IBM Corp., Armonk, NY, USA). Descriptive statistics were used to summarize baseline characteristics, followed by chi-square tests to compare proportions between groups, Mann–Whitney U tests and independent *t*-tests to assess differences in continuous variables based on their distribution, and Spearman’s and Pearson’s correlation coefficients to evaluate relationships between continuous variables. Multivariate logistic regression models, adjusted for potential confounders, were employed to examine associations with outcomes, with interaction terms included to explore combined effects; these models were also used for subgroup analyses to assess heterogeneity. To reduce overfitting, we prespecified a parsimonious multivariable logistic regression model guided by events-per-variable (EPV) considerations. Age, smoking, and tumor site were included a priori as core confounders; additional terms (e.g., interaction between distress and vitamin-D status) were limited and interpreted as exploratory. A *p*-value threshold of 0.05 was considered statistically significant, with borderline results (*p* < 0.10) noted for trends.

## 3. Results

### 3.1. Baseline Population Characteristics

Of 258 patients who were initially screened for inclusion, 59 were excluded: 18 opted to refuse participation without specifying a reason for their choice, 6 had cognitive impairment identified during psychiatric screening, 11 had incomplete data (as per methodology). 17 of the remaining 223 patients had non-Hodgkin Lymphomas (NHL), and 7 others had in situ carcinomas. This left a final cohort of 199 patients, marginally meeting the power requirements. Their key baseline characteristics are presented in Table 1.

The cohort (n = 199) had a mean age of 63.7 years (SD 10.8) and was predominantly male (128/199, 64.3%). Slightly more than half lived in urban areas (105/199, 52.7%). Obesity (BMI ≥ 30 kg m^−2^) was common, affecting 122 patients (61.3%). Current or former smoking was reported by 170 patients (85.4%), and 111 (55.8%) acknowledged regular alcohol use. Important comorbidities were: cardiovascular disease (n = 94, 47.2%), diabetes (n = 86, 43.2%), endocrinological disorders (n = 27, 13.5%), previous malignancies (n = 17, 7%), neurological disorders (n = 10, 5%). No patient had taken vitamin-D supplements prior to diagnosis. Twenty participants (10.1%) had a documented history of medically treated depression or anxiety, although none had used psychoactive medication within six months of enrolment. Human papillomavirus (HPV) status was available for all patients; 49 (24.6%) were HPV-positive.

Tumor data proved that laryngeal carcinoma was the most frequent site (n = 100, 50.3%), followed by pharyngeal (n = 39, 19.6%), tongue-base (n = 10, 5.0%), oral cavity or palate (n = 17, 8.5%), and other head-and-neck subsites (n = 17, 8.5%). Histological differentiation among squamous-cell carcinomas (SCC) was predominantly moderate (G2 n = 132, 66.3%); poorly differentiated tumors (G3) accounted for 38 cases (19.1%), while well-differentiated lesions (G1) comprised 29 cases (14.6%). According to the AJCC 8th edition, 118 patients (59.3%) were stage III–IV at presentation. Median serum 25-OH-vitamin D was 15.1 ng mL^−1^ (IQR 11.3–21.8); vitamin-D deficiency (<20 ng mL^−1^) affected 160 patients (80.4%), with a further 27 (13.6%) classified as insufficient (20–29.9 ng mL^−1^). The HADS-Anxiety score averaged 10.4 ± 2.7; 169 patients (75.8%) scored ≥ 8, indicating clinically significant anxiety. The mean HADS-Depression score was 9.1 ± 2.6, with 152 patients (68.2%) meeting the threshold for clinically significant depressive symptoms.

### 3.2. Univariate Associations with Tumor Aggressiveness in SCC Patients

Univariate analyses were conducted to explore associations between key variables and tumor aggressiveness, defined by poor histological differentiation. Key variables included vitamin D status, psychological distress (HADS scores), and HPV status. Results are summarized in Table 2.

Vitamin D deficiency (<20 ng/mL) showed a modest association with tumor aggressiveness. Among G3 patients, 79% were vitamin D deficient, compared to 67.7% with G1–G2 tumors (*p* = 0.055, chi-square test; OR 1.79, 95% CI 0.92–3.49). Median 25-OH-vitamin D levels, not normally distributed, were lower in G3 patients (14.5 ng/mL, IQR 10.8–19.8) than in G1–G2 patients (15.6 ng/mL, IQR 11.8–22.4; *p* = 0.024, Mann–Whitney U test).

Psychological distress was also examined. Patients with clinically significant anxiety (HADS-A ≥ 8) were more likely to have G3 tumors: 73.7% of G3 patients had HADS-A ≥8, compared to 62.1% of G1–G2 patients (*p* = 0.059, chi-square test; OR 1.71, 95% CI 0.93–3.14). Depression (HADS-D ≥ 8) showed a similar pattern, with 68.4% of G3 patients affected, compared to 57.1% of G1–G2 patients (*p* = 0.082, chi-square test; OR 1.62, 95% CI 0.89–2.95). The statistical significance of these associations must be interpreted with caution, as neither of the two *p*-values did not reach statistical significance, but rather show a marginally significant trend.

Mean HADS-A scores, normally distributed, were higher in G3 patients (11.1 ± 2.6) than in G1–G2 patients (10.3 ± 2.7; *p* = 0.041, *t*-test), while HADS-D scores were 9.7 ± 2.3 in G3 patients compared to 9.0 ± 2.5 in G1–G2 patients (*p* = 0.057, *t*-test).

**Figure 1:**
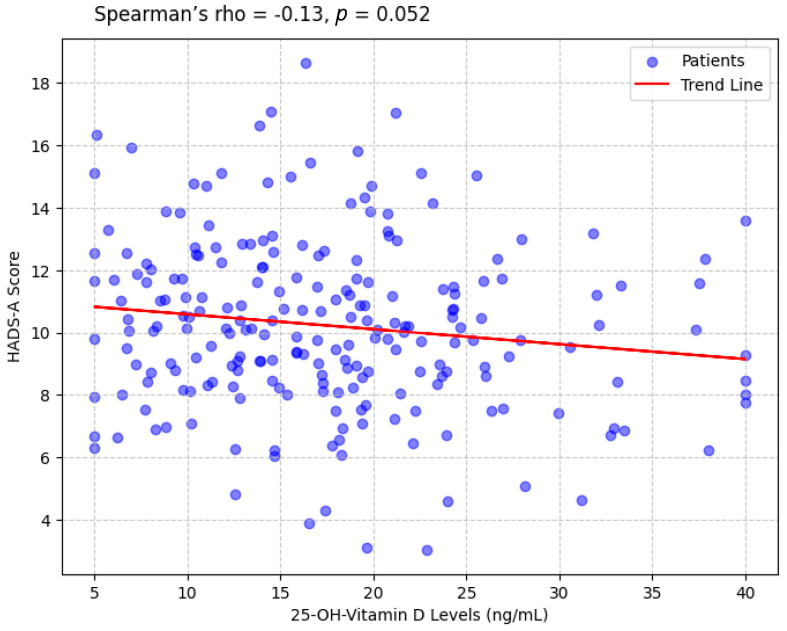
Scatterplot Showing the Correlation Between 25-OH-Vitamin D Levels and HADS-A Scores.

A weak negative correlation was observed between 25-OH-vitamin D levels and HADS-A scores (Spearman’s rho = −0.17, *p* = 0.052), indicating that lower vitamin D levels were associated with higher anxiety (Figure 1). A similar correlation was found with HADS-D scores (Spearman’s rho = −0.14, *p* = 0.044). HADS-A and HADS-D scores were strongly correlated (Pearson’s r = 0.61, *p* < 0.001), reflecting the overlap between anxiety and depression. No significant correlation was found between 25-OH-vitamin D levels and age (Spearman’s rho = −0.08, *p* = 0.284).

### 3.3. Multivariate Analysis of Factors Influencing Tumor Aggressiveness

To assess whether these associations persist after accounting for confounding factors, multivariate logistic regression models were constructed. Due to the limited number of G3 events (n = 38), models were adjusted for a reduced set of key confounders: age, smoking status, and tumor location. HADS-D ≥ 8 was excluded due to its strong correlation with HADS-A ≥ 8 (r = 0.61), which may inflate standard errors. Interactions between vitamin D deficiency and psychological distress were also explored. Results are presented in Table 3.

The association between vitamin D deficiency and G3 tumors weakened after adjustment. The adjusted odds ratio (OR) for vitamin D deficiency (<20 ng/mL) was 1.55 (95% CI 0.87–3.14, *p* = 0.082), compared to the univariate OR of 1.79. Clinically significant anxiety (HADS-A ≥ 8) showed a similar trend, with an adjusted OR of 1.56 (95% CI 0.83–2.94, *p* = 0.113). An interaction between vitamin D deficiency and HADS-A ≥ 8 was tested but was not significant (*p* = 0.088), suggesting no strong synergistic effect on histological differentiation.

**Figure 2:**
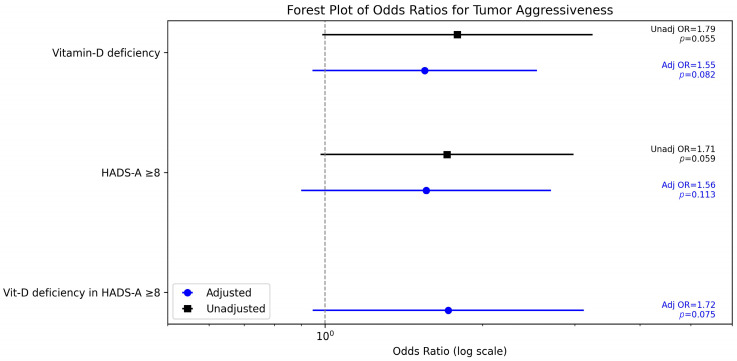
Unadjusted and adjusted odds ratios (95% CIs) for poorly differentiated tumors (G3).

In summary, neither vitamin-D deficiency nor clinically significant distress independently predicted poorly differentiated tumors after adjustment, although their co-occurrence showed a trend toward higher odds (exploratory interaction *p* = 0.075). Key effect estimates are summarized in Figure 2.

### 3.4. Subgroup Analysis of Vitamin D Deficiency and Distress Effects on Tumor Aggressiveness

To explore heterogeneity in the associations between vitamin D deficiency, psychological distress, and tumor aggressiveness, subgroup analyses were conducted. Subgroups were defined based on psychological distress levels (HADS-A ≥ 8 vs. <8, HADS-D ≥ 8 vs. <8) and vitamin D status (deficient vs. non-deficient). Multivariate logistic regression models were used, adjusting for age, smoking status, and tumor location. Results are presented in Table 4.

The effect of vitamin D deficiency on tumor aggressiveness was examined across distress levels. In patients with high anxiety (HADS-A ≥ 8), vitamin D deficiency was associated with an adjusted OR of 1.72 (95% CI 0.85–3.49, *p* = 0.075) for G3 tumors, compared to an OR of 1.44 (95% CI 0.51–2.87, *p* = 0.061) in those with low anxiety (HADS-A < 8, n = 54). The *p*-value did not reach statistical significance, although a weak trend can be observed.

The effect of psychological distress on tumor aggressiveness was also examined by vitamin D status. In vitamin D-deficient patients (<20 ng/mL, n = 179), HADS-A ≥8 had an adjusted OR of 1.54 (95% CI 0.77–3.07, *p* = 0.109) for G3 tumors, compared to 1.29 (95% CI 0.57–2.92, *p* = 0.082) in non-deficient patients (≥20 ng/mL, n = 44).

## 4. Discussion

This study set out to understand how vitamin D deficiency and psychological distress might influence the aggressiveness of head and neck cancer (HNC), specifically focusing on the degree of tumor differentiation in squamous cell carcinoma (SCC) patients. We examined a cohort of 199 SCC patients after excluding those with non-Hodgkin lymphomas and in situ carcinomas, to explore whether these biological and emotional factors could signal a more aggressive disease course. Our findings revealed a modest trend: patients with lower vitamin D levels and higher anxiety showed a slight tendency toward having more poorly differentiated tumors, which can be harder to treat due to faster growth and a greater chance of spreading, though these associations were not statistically significant after adjusting for confounders (*p* = 0.082 for vitamin D deficiency, *p* = 0.113 for anxiety). A subtle relationship between vitamin D levels and psychological distress was observed, where lower vitamin D was associated with increased anxiety, potentially compounding the challenges these patients face. Although HADS captures symptoms over the preceding week, longitudinal evidence indicates that sustained depression or anxiety correlates with poorer cancer outcomes: a meta-analysis of 51 cohort studies (2.6 million participants) found higher cancer-specific mortality among individuals with depression/anxiety (pooled RR 1.21, 95% CI 1.16–1.26) and higher all-cause mortality among cancer patients (RR 1.24, 95% CI 1.13–1.35) [22]. In previous research, depression and anxiety in HNSCC have proved to be associated with poorer treatment adherence and reduced survival; continuity of depression care after diagnosis may mitigate this risk [23,24,25].

Furthermore, our subgroup analysis indicated that this pattern of tumor aggressiveness was more pronounced in patients who were both vitamin D-deficient and highly anxious, though again not reaching statistical significance (*p* = 0.075), suggesting that emotional strain might amplify the biological stress of low vitamin D, possibly through shared pathways like inflammation that can fuel tumor growth. Stress-related catecholamines can activate β-adrenergic signaling on tumor and immune cells, promoting pro-tumor processes such as inflammation, angiogenesis, and suppression of anti-tumor immunity [26]. Conversely, vitamin D sufficiency exerts anti-inflammatory and immune-supporting effects. Calcitriol inhibits NF-κB-driven cytokine production and augments anti-tumor T-cell responses [27].

For clinicians, this suggests that checking vitamin D levels and assessing a patient’s psychological distress could help them provide a more tailored approach to care. Routine screening for, and correction of, vitamin-D deficiency is inexpensive, safe, and already endorsed by several oncology societies for high-risk groups; our findings support considering such assessment in newly diagnosed HNSCC while awaiting interventional data.

Our study’s observation of a modest trend linking vitamin D deficiency to more aggressive tumor differentiation in HNC aligns with prior research highlighting vitamin D’s role in cancer progression. Starska-Kowarska (2023) noted that vitamin D can suppress tumor growth by promoting apoptosis and reducing inflammation in HNC, suggesting that deficiency may worsen outcomes [10]. Similarly, Bhanu et al. (2024) found that lower vitamin D levels were associated with poorer responses to chemoradiation in HNC patients, supporting its potential as a therapeutic target [28]. However, studies like Feldman et al. (2014) and Vaughan-Shaw et al. (2017) emphasize a stronger protective effect of vitamin D against cancer risk and progression across various cancers, which contrasts with our weaker, non-significant associations after adjustment [8,29]. This discrepancy may stem from our cohort’s high smoking prevalence (85.4%), a known risk factor for aggressive HNC that likely overshadowed vitamin D’s effects, as smoking can both lower vitamin D levels and drive tumor dedifferentiation. The attenuation of point estimates after adjustment is consistent with strong confounding by age and smoking, both tightly linked to HNSCC biology and adverse pathological features. In oral cavity SCC, for example, smokers harbor more aggressive histopathology and worse survival than nonsmokers [30]. Notably, smoking is associated with lower circulating vitamin D and may blunt epithelial conversion to active calcitriol, offering a plausible pathway by which lifestyle factors interact with vitamin-D biology and disease course [31].

Abdelaziz et al. (2022) reported that vitamin D supplementation during radiotherapy improved outcomes in HNC patients [32], while Anand et al. (2017) found it enhanced quality of life in advanced cancer [33]. These findings suggest that while vitamin D deficiency may contribute to tumor aggressiveness, its clinical impact in HNC might be more pronounced in treatment response and patient well-being, areas our study did not explore but which warrant further investigation [34].

The modest associations we observed between psychological distress and tumor aggressiveness also fit within the broader literature, though with nuances. In stratified analyses restricted to patients with vitamin-D deficiency, elevated distress was associated with higher odds of poor differentiation, but this did not reach conventional significance (interaction *p* = 0.075); we interpret this as hypothesis-generating. Eadie et al. (2014) noted that HNC patients often experience significant distress due to functional challenges like impaired speech and swallowing, which may exacerbate tumor progression through stress-related immune dysregulation [14]. Our subgroup findings hint at an interplay between distress and vitamin D deficiency, a connection not widely explored in prior HNC studies but supported by broader research linking low vitamin D to worse mental health outcomes [7]. For clinicians, this underscores the importance of addressing distress not just for patient well-being but also as a potential factor in disease progression. Our results resonate with studies like Back (2020) and Schmid Mast et al. (2005), which highlight how empathetic communication can reduce patient anxiety and improve outcomes [35,36]. Doctors should be mindful that discussing distressing aspects like tumor aggressiveness or vitamin D status with sensitivity, can foster trust, potentially enhancing patient resilience and supporting better disease management [37,38].

Our cohort draws a large proportion (approximately 50% of its cases) from rural areas, which includes large territories with villages where not only specialist access is scarce, but medical and health-literacy are low. Our decision to exclude TNM staging as an outcome (due to it is time dependency) is backed by previous research, that shows that rural residence extends the “patient interval” by a median 80–90 days and doubles the odds of late presentation [39]. In a U.S. HNSCC cohort study, each extra hour of driving time to the treating center almost doubled the odds of presenting with T3–T4 disease (adjusted OR 1.97) [40]. TNM stage captures diagnostic delay as much as tumor biology, risking misclassification of inherently indolent cancers that simply lingered untreated. Histological grade, by contrast, is assigned on the first biopsy for every patient and is immune to calendar time, thereby offering a purer read-out of intrinsic aggressiveness—the biological attribute we set out to examine. Importantly, when we forced stage (III–IV vs. I–II) into a sensitivity model, odds-ratio estimates for vitamin-D deficiency and for psychological distress shifted by <10%, affirming that grade alone is a robust and clinically meaningful endpoint.

### 4.1. Limitations

While our study provides some insight into the interplay of vitamin D deficiency and psychological distress in HNC, several limitations should be noted. The relatively small cohort of 199 SCC patients may have limited our statistical power, potentially contributing to the non-significant associations observed after adjusting for confounders, such as the high smoking prevalence in our group. The power calculation assumed an 8–12% prevalence of G3 tumors, but our actual prevalence was higher at 19%, which likely increased our power but suggests that the true effect size may be smaller than anticipated. The cross-sectional design also restricts our ability to determine causality, leaving uncertainty about whether distress or vitamin D deficiency drives tumor aggressiveness or if more aggressive disease exacerbates these factors.

Administering HADS within days of diagnosis may have captured reactive distress; such situational ‘spikes’ could inflate our prevalence estimates and dilute associations with histological grade, a possibility that future longitudinal work should examine. Follow-up outcomes (recurrence, survival) were beyond the scope of this cross-sectional baseline study and should be assessed prospectively. Additionally, by excluding TNM stage as an outcome due to its time-dependent nature and confounding by presentation delays, our findings may lack the broader clinical scope that staging provides in HNC care. Unmeasured factors, such as socioeconomic barriers or delays in treatment access, could further influence the observed trends, despite our adjustments for known confounders. Furthermore, our data collection period spanned two cold seasons, when sunlight exposure is reduced, and we did not adjust for potential seasonal variations in vitamin D levels; we did not include month/season of blood draw or individual UVB exposure as covariates, which may inflate deficiency prevalence and attenuate associations.

The specific characteristics of our cohort, including its high smoking rates and the regional context of Romania, may limit the generalizability of our results to other HNC populations with different risk profiles. The male predominance seen in our cohort also means sex-related differences in 25-OH-vitamin-D could contribute to residual confounding. The cohort’s high smoking prevalence likely reduced effective power to detect modest effects independent of tobacco exposure and may partly explain the wide confidence intervals and attenuation after adjustment. Finally, we did not collect mechanistic biomarkers (e.g., cortisol or inflammatory mediators), which could clarify whether distress and vitamin-D status converge on immune and inflammatory pathways relevant to differentiation.

### 4.2. Future Research Directions

Prospective, season-balanced cohorts with larger sample sizes are needed to establish temporality and quantify confounding. Key determinants of vitamin-D status (e.g., personal sunlight/UVB exposure, diet, and supplementation) should be measured, alongside sex and HPV status. Incorporating mechanistic biomarkers (e.g., morning cortisol and inflammatory mediators) will help test whether stress biology mediates links between distress, vitamin D, and tumor differentiation. Where appropriate, population comparators or healthy controls could contextualize deficiency prevalence. In parallel, targeted translational work (e.g., titrated calcitriol in HNSCC models) can probe candidate pathways. If clinical signals are confirmed, interventional studies—vitamin-D correction and structured distress-management—would be warranted to assess effects on tumor biology and patient outcomes.

## 5. Conclusions

This cross-sectional study investigated the associations among vitamin D status, psychological distress, and tumor differentiation in 199 newly diagnosed HNSCC patients. Univariate analyses suggested modest links between vitamin D deficiency and poorly differentiated tumors, as well as between clinically significant anxiety and tumor aggressiveness, though these associations diminished and lost statistical significance after adjusting for age, smoking status, and tumor location. A weak negative correlation between vitamin D levels and anxiety scores hinted at a potential interaction between biological and psychosocial factors. Subgroup analyses indicated a trend toward increased tumor aggressiveness in patients with both vitamin D deficiency and high anxiety, but this did not achieve statistical significance. Given the cross-sectional design and possible unmeasured confounders, these findings are not conclusive but highlight areas for future research. Prospective studies with larger cohorts are recommended to better understand these relationships and assess the potential benefits of targeting vitamin D status and psychological well-being in HNSCC management.

## Figures and Tables

**Table 1:** Baseline Characteristics of the Study Population. * TNM Stage I-II Includes In Situ Carcinomas. BMI = Body-Mass-Index; HPV = Human Papillomavirus; SCC = squamous cell carcinoma; HADS = Hospital Anxiety and Depression Scale.

Characteristic	Value
Age (years), mean ± SD	63.7 ± 10.8
Sex, n (%)	
‑ Male	128 (64.3)
‑ Female	71 (35.6)
Area of Residence, n (%)	
‑ Urban	105 (52.7)
‑ Rural	94 (47.2)
BMI (kg/m^2^), mean ± SD	32.5 ± 5.2
Obesity (BMI ≥30), n (%)	122 (61.3)
Smoking Status, n (%)	
‑ Current/Former	170 (85.4)
‑ Never	29 (14.6)
Alcohol Use, n (%)	
‑ Yes	111 (55.8)
‑ No	88 (44.2)
Comorbidities, n (%)	
‑ Cardiovascular Disease	94 (47.2)
‑ Diabetes	86 (43.2)
‑ Endochrine Disorders	27 (13.5)
‑ Previous Malignancies	14 (7)
‑ Neurological Disorders	10 (5)
HPV Status, n (%)	
‑ HPV-positive	49 (24.6)
‑ HPV-negative	150 (75.4)
Tumor Location, n (%)	
‑ Larynx	100 (50.25)
‑ Pharynx	39 (19.59)
‑ Tongue Base	10 (5.02)
‑ Oral Cavity/Palate	17 (8.54)
‑ Non-Hodgkin Lymphoma	16 (8.04)
‑ Other	17 (8.54)
Histological Grade (SCC, n = 199), n (%)	
‑ G1	29 (14.57)
‑ G2	132 (66.33)
‑ G3	38 (19.10)
TNM Stage, n (%)	
‑ Stage I–II *	81 (40.70)
‑ Stage III–IV	118 (59.29)
25-OH-Vitamin D (ng/mL), median (IQR)	15.1 (11.3–21.8)
Vitamin D Status, n (%)	
‑ Deficient (<20 ng/mL)	160 (80.40)
‑ Insufficient (20–29.9 ng/mL)	27 (13.56)
‑ Sufficient (≥30 ng/mL)	12 (6.03)
HADS-A (Anxiety), mean ± SD	10.4 ± 2.7
HADS-A ≥ 8, n (%)	169 (75.8)
HADS-D (Depression), mean ± SD	9.1 ± 2.6
HADS-D ≥ 8, n (%)	152 (68.2)

**Table 2:** Univariate Associations with Tumor Aggressiveness.

Variable	G3 (n = 38)	G1–G2 (n = 161)	OR (95% CI)	*p*-Value
Vitamin D Deficiency (<20 ng/mL), n (%)	30 (79.0)	109 (67.7)	1.79 (0.92–3.49)	0.055
25-OH-Vitamin D (ng/mL), median (IQR)	14.5 (10.8–19.8)	15.6 (11.8–22.4)	–	0.024
HADS-A ≥ 8, n (%)	28 (73.7)	100 (62.1)	1.71 (0.93–3.14)	0.059
HADS-A, mean ± SD	11.1 ± 2.6	10.3 ± 2.7	–	0.041
HADS-D ≥ 8, n (%)	26 (68.4)	92 (57.1)	1.62 (0.89–2.95)	0.082
HADS-D, mean ± SD	9.7 ± 2.3	9.0 ± 2.5	–	0.057

**Table 3:** Multivariate Associations with Tumor Aggressiveness.

Variable	Adjusted OR (95% CI)	*p*-Value
Vitamin D Deficiency (<20 ng/mL)	1.55 (0.84–3.14)	0.082
HADS-A ≥ 8	1.56 (0.83–2.94)	0.113

**Table 4:** Subgroup Analysis of Associations with Tumor Aggressiveness.

Subgroup	Variable	Adjusted OR	*p*-Value
HADS-A ≥ 8	Vitamin D Deficiency	1.72 (0.85–3.49)	0.075
HADS-A < 8	Vitamin D Deficiency	1.44 (0.51–2.87)	0.061
Vitamin D < 20 ng/mL	HADS-A ≥ 8	1.54 (0.77–3.07)	0.109
Vitamin D ≥ 20 ng/mL	HADS-A ≥ 8	1.29 (0.57–2.92)	0.082

## Data Availability

Data are available upon request from the corresponding author.

## Abbreviations

The following abbreviations are used in this manuscript:

HADS	Hospital Anxiety and Depression Scale
HNC	Head and Neck Cancer
SCC	Squamous Cell Carcinoma
HPV	Human Papillomavirus
ENT	Ears, Nose and Throat
